# Anti-vascular effects of the cytosolic phospholipase A2 inhibitor AVX235 in a patient-derived basal-like breast cancer model

**DOI:** 10.1186/s12885-016-2225-1

**Published:** 2016-03-07

**Authors:** Eugene Kim, Hanna Maja Tunset, Jana Cebulla, Riyas Vettukattil, Heidi Helgesen, Astrid Jullumstrø Feuerherm, Olav Engebråten, Gunhild Mari Mælandsmo, Berit Johansen, Siver Andreas Moestue

**Affiliations:** Department of Circulation and Medical Imaging, Faculty of Medicine, Norwegian University of Science and Technology, P.O. Box 8905, 7491 Trondheim, Norway; Department of Biology, Norwegian University of Science and Technology, Realfagbygget, 7491 Trondheim, Norway; Department of Tumor Biology, Institute for Cancer Research, The Norwegian Radium Hospital, Oslo University Hospital, P.O. Box 4953 Nydalen, Oslo, 0424 Norway

**Keywords:** Angiogenesis, Breast cancer, Choline metabolism, Cytosolic phospholipase A2, Dynamic contrast enhanced MRI, Micro-CT, Prostaglandin E2, Targeted therapy

## Abstract

**Background:**

Group IVA cytosolic phospholipase A2 (cPLA2α) plays an important role in tumorigenesis and angiogenesis. It is overexpressed in basal-like breast cancer (BLBC), which is aggressive and usually triple-negative, making it unresponsive to current targeted therapies. Here, we evaluated the anti-angiogenic effects of a specific cPLA2α inhibitor, AVX235, in a patient-derived triple-negative BLBC model.

**Methods:**

Mice bearing orthotopic xenografts received i.p. injections of AVX235 or DMSO vehicle daily for 1 week and then every other day for up to 19 days. Six treated and six control mice were terminated after 2 days of treatment, and the tumors excised for high resolution magic angle spinning magnetic resonance spectroscopy (HR MAS MRS) and prostaglandin E2 (PGE2) enzyme immunoassay (EIA) analysis. A 1-week imaging study was performed on a separate cohort of mice. Longitudinal dynamic contrast enhanced (DCE)-MRI was performed before, after 4 days, and after 1 week of treatment. The mice were then perfused with a radiopaque vascular casting agent, and the tumors excised for micro-CT angiography. Subsequently, tumors were sectioned and stained with lectin and for Ki67 or α-smooth muscle actin to quantify endothelial cell proliferation and vessel maturity, respectively. Partial least squares discriminant analysis was performed on the multivariate HR MAS MRS data, and non-parametric univariate analyses using Mann–Whitney U tests (α = 0.05) were performed on all other data.

**Results:**

Glycerophosphocholine and PGE2 levels, measured by HR MAS MRS and EIA, respectively, were lower in treated tumors after 2 days of treatment. These molecular changes are expected downstream effects of cPLA2α inhibition and were followed by significant tumor growth inhibition after 8 days of treatment. DCE-MRI revealed that AVX235 treatment caused a decrease in tumor perfusion. Concordantly, micro-CT angiography showed that vessel volume fraction, density, and caliber were reduced in treated tumors. Moreover, histology showed decreased endothelial cell proliferation and fewer immature vessels in treated tumors.

**Conclusions:**

These results demonstrate that cPLA2α inhibition with AVX235 resulted in decreased vascularization and perfusion and subsequent inhibition of tumor growth. Thus, cPLA2α inhibition may be a potential new therapeutic option for triple-negative basal-like breast cancer.

## Background

Basal-like breast cancer (BLBC), which represents ~15 % of all breast cancers [1], is an aggressive molecular subtype of the disease associated with poor prognosis [1, 2]. Most BLBCs are triple-negative [3] (lacking expression of estrogen receptor, progesterone receptor, and human epidermal growth factor receptor 2) and thus unresponsive to currently available targeted therapies. Hence, new molecular targets for treatment are called for. Interestingly, it has been shown that BLBC patient samples and patient-derived xenografts overexpress the gene *PLA2G4A* [4, 5], which encodes group IVA cytosolic phospholipase A2 (cPLA2α), indicating increased activity and an important functional role of the enzyme in this subtype.

There is growing evidence of the involvement of cPLA2α in tumorigenesis and angiogenesis in various types of cancer [6, 7], and cPLA2α inhibition has been shown to reduce tumor development and growth in several animal models [8–11]. Cytosolic PLA2α is the only PLA2 with specificity for arachidonic acid (AA)-containing phospholipids [12]. Upon activation, cPLA2α cleaves such membrane phospholipids to release AA and lysophospholipids. These molecules and their metabolites can produce a plethora of biological effects, such as transcriptional regulation, remodeling of phospholipid metabolism, inflammation, and angiogenesis. Lysophosphatidylcholine is a lipid second messenger that can activate Akt and mitogen-activated protein kinase and induce endothelial cell proliferation by transactivation of vascular endothelial growth factor (VEGF) receptor 2 [13–15]. Eicosanoids, enzymatic metabolites of AA, are bioactive lipid signaling molecules that act in an autocrine and paracrine manner. One of the principal eicosanoids resulting from cPLA2α activation, prostaglandin E2 (PGE2), is a pro-inflammatory, mitogenic, anti-apoptotic, and pro-angiogenic molecule [16]. The importance of increased PGE2 levels in various cancer types [17], including breast cancer [18–20], has been established.

Based on this, we aimed to characterize the effect of cPLA2α inhibition on tumor growth and vasculature in a patient-derived BLBC xenograft model [21]. The cPLA2α-specific inhibitor used, AVX235 (Avexxin AS, Trondheim, Norway), is a thiazolyl ketone (methyl 2-(2-(4-octylphenoxy)acetyl)thiazole-4-carboxylate) that was originally developed as an anti-inflammatory drug [22]. It has previously been tested in a collagen-induced arthritis model, with no adverse effects and an efficacy comparable to reference drugs in pertinent doses [22]. In our study, downstream metabolites of cPLA2 α were quantified with enzyme immunoassays (EIA) and ex vivo ^1^H high-resolution magic angle spinning magnetic resonance spectroscopy (HR MAS MRS) to verify inhibition of cPLA2α by AVX235, and the tumor growth response to AVX235 was measured. Longitudinal in vivo magnetic resonance imaging (MRI) was used to measure changes in tumor vascular function, and ex vivo micro-computed tomography (μCT) was used to characterize the effects on vascular morphology. Immunohistochemistry was performed to evaluate cancer and endothelial cell proliferation and vessel maturity.

## Methods

### Animal model

The patient-derived MAS98.12 basal-like/triple-negative breast cancer xenograft model was established and maintained as described in [21]. For this study, MAS98.12 tumor fragments were bilaterally implanted into the thoracic mammary fat pads of female Hsd:Athymic Nude-Foxn1^*nu*^ mice. The animals were kept under pathogen-free conditions. Housing conditions included temperature between 19 and 22 °C, humidity between 50 and 60 %, 20 air changes/h and a 12 h light/dark cycle. The animals were fed RM1 diet (Scanbur BK, Karlslunde, Denmark) and distilled tap water ad libitum. The drinking water was supplemented with 17-β-estradiol at a concentration of 4 μg/ml in order to achieve the same conditions as in [21], although it has been shown to have no influence on the growth of these estrogen receptor-negative tumors [23].

### Study design

For the tumor growth study, each mouse was randomly assigned to treatment or control groups when the diameter of the larger of its two tumors reached ~6 mm. Tumor volume was calculated from caliper measurements as 1/2 × length × width^2^. The mice received either 30 mg/kg AVX235 dissolved in 50 μl of 100 % DMSO (treatment groups) or matched volumes of DMSO (control groups) by intraperitoneal (i.p.) injection for 2 days (*n* = 6 for each group) or 19 days (*n* = 6 for each group). A lower dose was used in the longer tumor growth study than in the imaging study (see below) due to a limited supply of the drug and to reduce the risk of adverse effects. Injections were administered daily for the first week, then every second day in the 19-day groups to avoid adverse effects of DMSO. The mice were weighed at least twice a week and were checked daily by trained personnel. The length of the study was limited by the tumor growth rate in the control group. Animals were euthanized by cervical dislocation after two or 19 days, or when the humane endpoint of a maximum allowed tumor diameter of 12 mm was reached (day 16 for two mice in the 19-day control group). Immediately after excision, one tumor (or one tumor half, when only one tumor had established in the animal) from each animal was preserved in liquid nitrogen for PGE2 EIA and ^1^H HR MAS MRS, and the other in neutral buffered formalin (NBF) for subsequent histological analyses.

For the imaging study, mice were randomly assigned to treatment or control groups when the long axis of the larger tumor reached ~8 mm as measured by calipers. The treatment group (*n* = 9) received 45 mg/kg of AVX235 daily and the control group (*n* = 8) received daily volume-matched doses of DMSO (50 μL) by i.p. injections. Animals were weighed daily and imaged with MRI on days 0 (immediately prior to the first dosing), 4, and 7. The mice were then euthanized by pentobarbital overdose and perfusion fixation, and the tumors excised for μCT imaging and histology. Details of the methods are provided below.

### PGE2 EIA

RNA was purified from tumor tissue using a standard RNA isolation kit (Qiagen RNeasy mini kit, Cat. no. 74104; Qiagen, Limburg, The Netherlands). Tumor samples of 12 ± 3 mg (mean ± SD) were cut from frozen tumors, added to ice-cold lysis buffer with 10 μM indomethacin and 10 μl/ml β-mercaptoethanol, and homogenized using a Precellys 24 (Bertin Corp., Washington, D.C., USA) at 5200 rpm for 20 s. Homogenized samples were treated according to the kit manual. RNA levels were measured using a Nanodrop 2000 (Thermo Scientific, Waltham, MA, USA) and used to normalize PGE2 levels. For PGE2 analysis, the flow-through of the ethanolic fraction from the RNA isolation kit was collected and stored at -80 °C and at -20 °C prior to use (less than 3 months at -20 °C). PGE2 levels in the flow-through samples were determined using a PGE2 EIA kit (Item no. 514010, Cayman Chemical, Ann Arbor, MI, USA) and a Multiskan Ascent plate reader (MTX Lab Systems, Inc., Vienna, VA, USA). The samples were spun at 8000 g for 10 min, and the supernatant diluted with assay buffer (typically between 1:25 and 1:100) to ensure readings were within the recommended 20–80 % transmittance range. Any diluted sample that was out of range was excluded. Two to seven technical replicate measurements of each flow-through sample were acquired. Biological replicates (different samples from the same tumor) were analyzed for four tumors. A four-parameter logistic model was fit to the absorbance data to determine PGE2 levels. In order to eliminate errors from weighing of tumor samples and to correct for necrotic or adipose tissue, PGE2 levels were normalized to the RNA levels isolated from the same sample.

### HR MAS MRS

For HR MAS MRS, frozen xenograft tissue (6.4 ± 3.0 mg [mean ± SD]) from 1 tumor per animal (*n* = 6 per group) was cut to fit into 30 μl disposable inserts (Bruker BioSpin, Ettlingen, Germany) containing 3 μl of 25 mM sodium formate in D_2_O. HR MAS MR spectra were obtained using a Bruker AVANCE DRX-600 spectrometer with a ^1^H/^13^C HR MAS probe (Bruker BioSpin). Samples were spun at 5 kHz at 5 °C, and a Carr-Purcell-Meiboom-Gill experiment (cpmg, Bruker; acquisition time = 3.1 s, sweep width = 20 ppm, 256 scans) was performed for all samples. Post-processing of spectra included 0.3 Hz exponential line broadening and baseline correction. Data analysis was performed with MATLAB (Version 7.9.0; The Math Works, Natick, MA, USA). Spectra were mean normalized to minimize differences in the sample weight. Supervised partial least squares discriminant analysis (PLS-DA) was performed (PLS_Toolbox v5.8.3, Eigenvector Research, Manson, WA, USA) to classify tumor samples as control or treated based on their spectra, and variable importance on projection (VIP) scores computed to determine the influence of each metabolite on the classification [24, 25].

### In vivo MRI

The larger of the bilateral tumors were imaged immediately prior to the start of treatment (day 0) and again on days 4 and 7. Imaging was performed on a 7.05 T horizontal bore MRI system (Bruker Biospin) using an 86 mm excitation volume coil and a quadrature receiver surface coil. The mice were anesthetized with isoflurane (2–2.5 % in 70 % air/30 % O_2_) during the MRI experiments. The isoflurane level was adjusted as needed to maintain a respiration rate of ~50 breaths/min, and body temperature was maintained at 37 °C using a small animal monitoring and gating system (Model 1030, SAII, Stony Brook, NY, USA).

MR images were acquired using the following sequences:

1. High-resolution 2D rapid acquisition with relaxation enhancement (RARE): effective echo time (TE_eff_) = 69 ms; repetition time (TR) = 1500 ms, RARE factor = 16, number of averages (NA) = 4; matrix = 256 × 192, zero-padded to 256 × 256.
2. 2D RARE with variable repetition times for baseline T1 measurement: TE_eff_ = 13 ms; TR = 225, 500, 1500, 3000, 6000, 12000 ms; RARE factor = 2; matrix = 64 × 48, zero-padded to 64 × 64.
3. Dynamic contrast enhanced (DCE)-MRI (2D RARE): TE_eff_ = 7.5 ms; TR = 300 ms; RARE factor = 4; matrix =  64 × 64; temporal resolution = 4.8 s, 200 images. An intravenous bolus injection of 0.3 mmol/kg of gadodiamide (Omniscan, GE Healthcare, Oslo, Norway) was administered via the tail vein after the tenth baseline image.

All scans were acquired with the same geometry: field of view = 20 × 20 mm; slice thickness = 0.6 mm, interslice gap = 0.3 mm, 4 coronal slices.

Tumor regions of interest were manually drawn on the high-resolution RARE images and then down-sampled to the resolution of the other images to mask out non-tumor tissue from the analysis. Maps of the initial area under the curve during the first minute after contrast injection (AUC_1min_) and the relative signal intensity (normalized to pre-contrast values) at 1 min post-contrast (RSI_1min_) were calculated voxel-wise from the dynamic signal enhancement time curves. To ensure that only perfused, viable tumor was included in the analysis, non-enhancing voxels in which RSI_1min_ < 1.5 were excluded. The fraction of enhancing voxels (FEV) was calculated for each tumor at each time point.

### Ex vivo μCT

Immediately after the final MRI examination, the mice were euthanized by pentobarbital overdose followed by intracardial perfusion with 20 ml each of saline, NBF, and finally Microfil® (Flow Tech, Inc., Carver, MA; USA). After allowing the Microfil to cure for 60 min, the tumors were excised and stored in NBF at 4 °C for 48 h. The tumors were then immersed sequentially in 30, 50, and 70 % ethanol at 4 °C for 24 h each. Then the tumors were imaged on a Bruker Skyscan 1176 μCT system (Bruker microCT, Kontich, BE) using the following parameters: 50 kV, 400 μA, 0.5 mm Al filter, 1020 ms exposure, 0.36° rotation step, 8 averages, 9 μm isotropic voxels. Images were reconstructed using the Feldkamp filtered back-projection algorithm. After μCT, the tumors were cut in half approximately along the planes of the in vivo MRI slices and then embedded in paraffin for histological analysis.

Blood vessels were segmented from the native μCT images in Fiji [26], an open-source image processing package, using a Hessian-based filtering method described in [27]. Tumor fractional blood volumes (FBV) were calculated by dividing the volumes of the segmented vessels by the tumor volumes. The Local Thickness plugin in Fiji (R. Dougherty, OptiNav, Inc., Bellevue, WA, USA) was used to compute vessel calibers (VC). The Exact Euclidean Distance Transform (3D) plugin in Fiji was used to calculate the distance between each non-vessel voxel in the tumor and the nearest vessel, i.e., to generate “distance to nearest vessel” (DNV) maps.

### Histology

Sections 4 μm thick were cut from the center of each tumor. Sections were double stained using lectin (*Griffonia simplicifolia* lectin I, Vector Laboratories, Burlingame, CA, USA) for endothelial cells and either anti-Ki67 (monoclonal rabbit anti-human Ki67 [SP6] with cross-reactivity to mouse; Abcam, Cambridge, United Kingdom) as a proliferation marker or anti-α-smooth muscle actin (monoclonal mouse anti-human α-SMA; Dako, Glostrup, Denmark) as a pericyte marker.

For each lectin/Ki67-stained section, non-overlapping random fields were acquired across the viable regions of the entire section at 40× on an Olympus BX41 microscope and saved as RGB TIFF images. Using a custom MATLAB script, the RGB images were converted to HSV (hue, saturation, value), and Ki67-positive and lectin-positive areas were segmented based on hue and saturation using the same manually determined set of thresholds for every image. The segmented images were used to count the number of Ki67-positive proliferating cells and compute the lectin-positive vessel area fraction. Overlapping Ki67-positive nuclei were separated using marker-based watershed segmentation. Proliferating endothelial cells were defined as Ki67-positive nuclei found within a lectin-positive vessel. For each field, the number of proliferating endothelial cells was normalized to the lectin-positive vessel area. A pathologist and a researcher experienced in evaluating such double stained sections were consulted to ensure that the automated algorithm was consistent with visual evaluation.

For each lectin/α-SMA-stained tumor section, non-overlapping random fields were acquired across the viable regions of the entire section at 10×, and α-SMA-positive and lectin-positive areas were segmented from the HSV images as described above. Lectin-positive vessels that were at least 5 μm away from the nearest α-SMA-positive pixel were identified, and the ratio of the area of these α-SMA-negative vessels to the total lectin-positive vessel area was computed for each tumor section. This lectin^α-SMA−^ area fraction was used as a measure of vessel immaturity.

### Statistical analysis

A permutation test was performed (1000 permutations) to evaluate the significance of the PLS-DA model [28]. Two-tailed Mann–Whitney U tests were performed to compare the control and treated tumors based on the following: 1) normalized tumor volume, 2) PGE2/RNA ratio, 3) tumor-wise median values of DCE-MRI parameters; 4) μCT-measured FBV, VC, and DNV; and 5) immunohistochemistry-derived measures of proliferation and vessel maturity. For all tests, α = 0.05. Values are presented as median ± median absolute deviation.

### Ethics approval

All procedures and experiments involving animals were approved by the Norwegian Animal Research Authority and carried out according to the European Convention for the Protection of Vertebrates used for Scientific Purposes.

## Results

### AVX235 reduces levels of cPLA2α downstream products

**Fig. 1:**
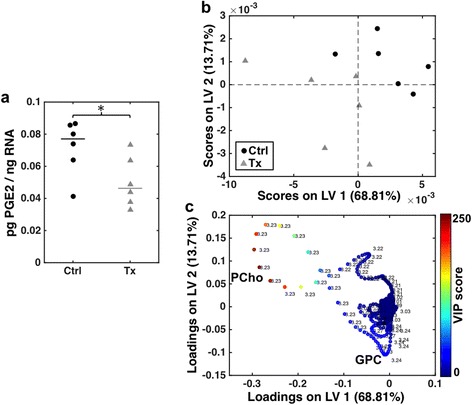
AVX235 effects downstream metabolites of cPLA2α. **a** After 2 days of AVX235 treatment, samples from treated mice (*grey triangles*) had significantly lower PGE2 levels compared to controls (*black dots*). PGE2 levels are normalized to RNA levels. Each symbol indicates the mean of 2–7 replicate measurements of the same tumor sample and/or different samples from the same tumor. Horizontal lines indicate group medians. * *p* < 0.05, two-tailed Mann–Whitney U test. **b-c** PLS-DA of HR MAS MR spectra from 2-day samples. **b** Scores plot showing separation of control and treated samples (*n* = 6 per group). **c** Loadings plot of the first two latent variables (LV1 and LV2) in the PLS-DA model. The VIP scores show that the most influential metabolites are PCho at 3.23 ppm and GPC at 3.24 ppm

The levels of PGE2 in tumor tissue were measured by EIA as a downstream biomarker of cPLA2α inhibition by AVX235 [22]. After 2 days of treatment, there were significantly lower PGE2 levels in tumor tissue from the treated mice (Tx) compared to controls (Ctrl): 0.046 ± 0.011 vs. 0.077 ± 0.009 pg PGE2/ng RNA, *p* = 0.041 (Fig. 1a). However, at day 19, there was no difference in PGE2 levels between the treated and control groups (0.046 ± 0.017 vs. 0.044 ± 0.002, *p* = 0.589), and 19-day controls contained significantly less PGE2 than 2-day controls (*p* = 0.026).

Cytosolic PLA2α is a mediator of choline metabolism, so ^1^H HR MAS MRS was performed to measure relative levels of choline-containing compounds to further verify that AVX235 inhibited cPLA2α activity. A clear separation of treatment and control groups after 2 days of AVX235 treatment was demonstrated by the PLS-DA scores plot (Fig. 1b), with a specificity and sensitivity of 83 %, and *p* = 0.004 by a permutation test. The loadings plot and VIP scores show that these changes were mainly attributed to higher phosphocholine (PCho, 3.23 ppm) and lower glycerophosphocholine (GPC, 3.24 ppm) levels in treated samples (Fig. 1c). After 19 days of treatment, significant differences between the groups did not persist, although a trend of lower GPC was observed in treated tumors (data not shown).

### AVX235 inhibits tumor growth

**Fig. 2:**
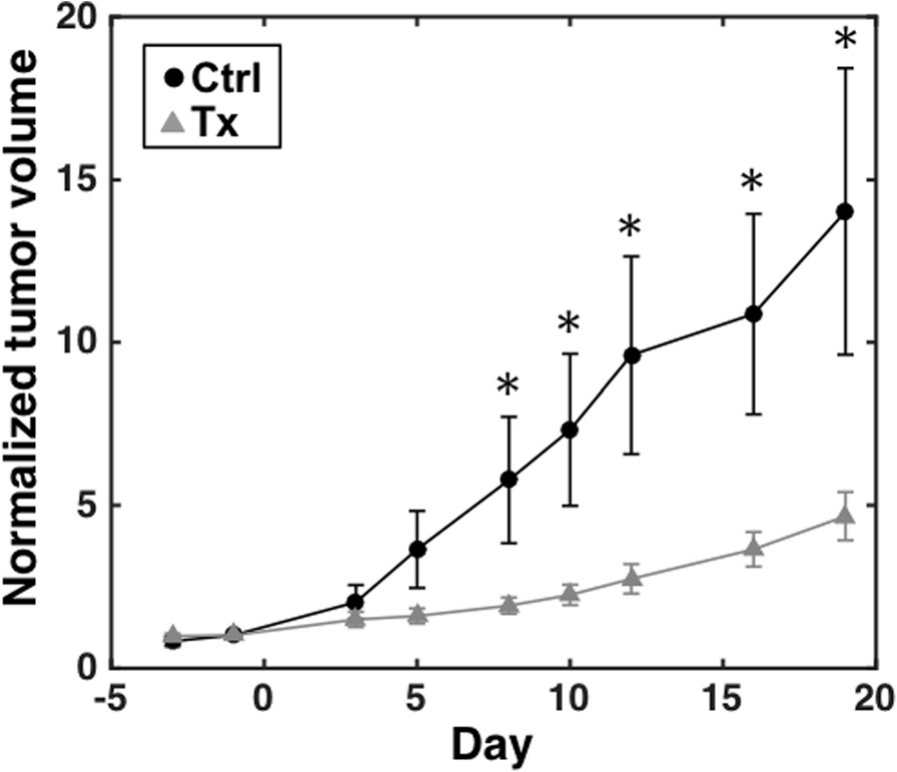
AVX235 inhibits tumor growth. 30 mg/kg AVX235 or vehicle (DMSO) alone was injected i.p. in MAS98.12 mice (daily for the first week, then every second day). The mean normalized tumor volumes are shown as black dots for control tumors (*n* = 10) and grey triangles for treated tumors (*n* = 11). Error bars represent the standard errors of the means. * *p* < 0.05, two-tailed Mann–Whitney U test, control vs. treated group on the same day

Tumor volumes were calculated from caliper measurements for up to 19 days to assess the effect of cPLA2α inhibition on the growth of BLBC tumor xenografts. Before the start of treatment, the median tumor volume was 69 ± 23 mm^3^ in the treatment group (*n* = 11) and 79 ± 52.5 mm^3^ in the control group (*n* = 10, *p* = 0.173 treatment vs. control at day -1). After 8 days of treatment, the tumor volumes normalized to day -1 values were significantly smaller in the treated group than in the control group (*p* = 0.029, Fig. 2). The difference in tumor volume between the groups remained significant throughout the study. After 19 days of treatment, the median treated tumor volume was 239 ± 91 mm^3^ compared to 626 ± 392 mm^3^ (*n* = 7) for controls (*p* = 0.017). The corresponding normalized volumes were 5.09 ± 2.02 and 8.00 ± 3.91 (*p* = 0.020). Two mice in the control group were terminated on day 16 due to unacceptable tumor burden (tumor diameter > 12 mm). No adverse effects (weight loss, physical appearance, or behavior) of AVX235 administration were observed.

In the imaging cohort, median tumor volumes were 192 ± 20 mm^3^ in the treatment group (*n* = 9) and 176 ± 37 mm^3^ in the control group (*n* = 8) on day 0 (*p* = 0.743). At the end of the 1-week study, the difference in tumor volumes between treatment and control groups (374 ± 80 mm^3^ vs. 389.5 ± 103.5 mm^3^) remained insignificant (*p* = 0.481), as did the difference in normalized tumor volumes (2.07 ± 0.20 vs. 2.54 ± 0.18, *p* = 0.200).

### cPLA2α inhibition reduces tumor perfusion

**Fig. 3:**
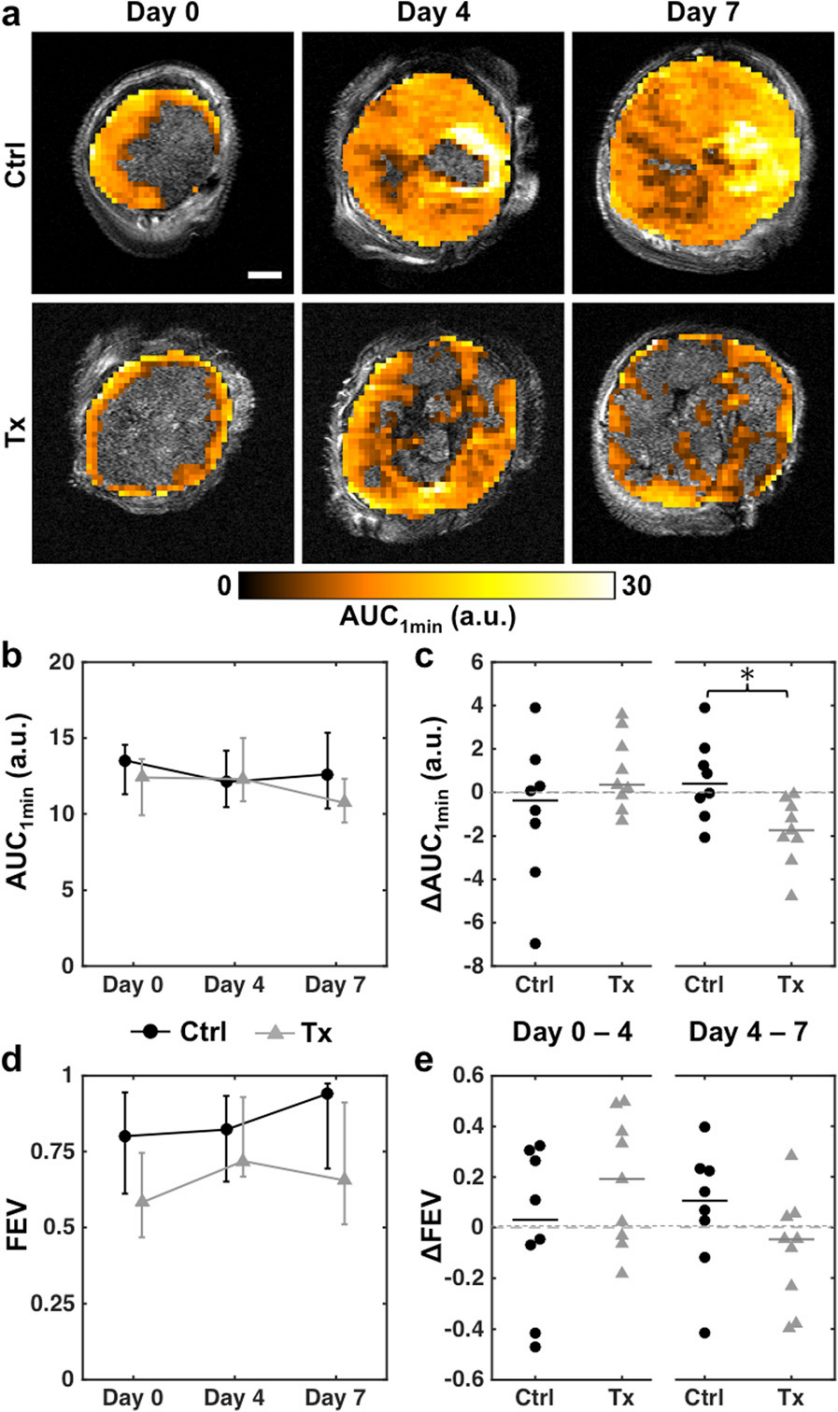
cPLA2α inhibition effects in vivo tumor perfusion measured by DCE-MRI. **a** Longitudinal AUC_1min_ maps from representative control and treated tumors, overlaid on their corresponding high-resolution anatomical images. Only enhancing voxels (voxels in which RSI_1min_ > 1.5) are displayed. Scale bar = 2 mm. **b** Group medians of tumor-wise median AUC_1min_ values of control (*black dots*) and treated (*grey triangles*) tumors. Error bars indicate interquartile ranges. **c** Dot plot of the changes in tumor-wise median AUC_1min_ values from day 0 to 4 and from day 4 to 7. Horizontal lines indicate group medians. **d**-**e** Analogous plots for FEV. * *p* < 0.05, two-tailed Mann–Whitney U test

Several cPLA2α-derived molecules such as lysophospholipids and PGE2 are recognized as modulators of angiogenic signaling [14, 16]. To assess the impact of AVX235 on tumor vascular function, DCE-MRI was performed to measure tumor perfusion in vivo. Figure 3a shows slices of the AUC_1min_ maps from representative control and treated tumors at each time point. Only enhancing voxels of the maps are shown overlaid on their respective high-resolution anatomical images. The pre-treatment images show characteristic enhancing tumor rims and non-enhancing cores. In control tumors, there was no clear longitudinal trend in the median AUC_1min_ (Fig. 3b-c). In the treated group, the median AUC_1min_ increased from day 0 to 4 in six of nine tumors and subsequently decreased from day 4 to 7 in all tumors (Fig. 3c). Similarly, FEV initially increased in six treated tumors and later decreased in six treated tumors (Fig. 3e). In contrast, FEV increased from day 4 to 7 in six of eight control tumors. While there were no significant differences in median AUC_1min_ or FEV between control and treated tumors at any time point (Fig. 3b,d), the change in AUC_1min_ from day 4 to 7 was significantly different between groups (*p* = 0.011, Fig. 3c).

### cPLA2α inhibition decreases tumor vascularization and vessel caliber

**Fig. 4:**
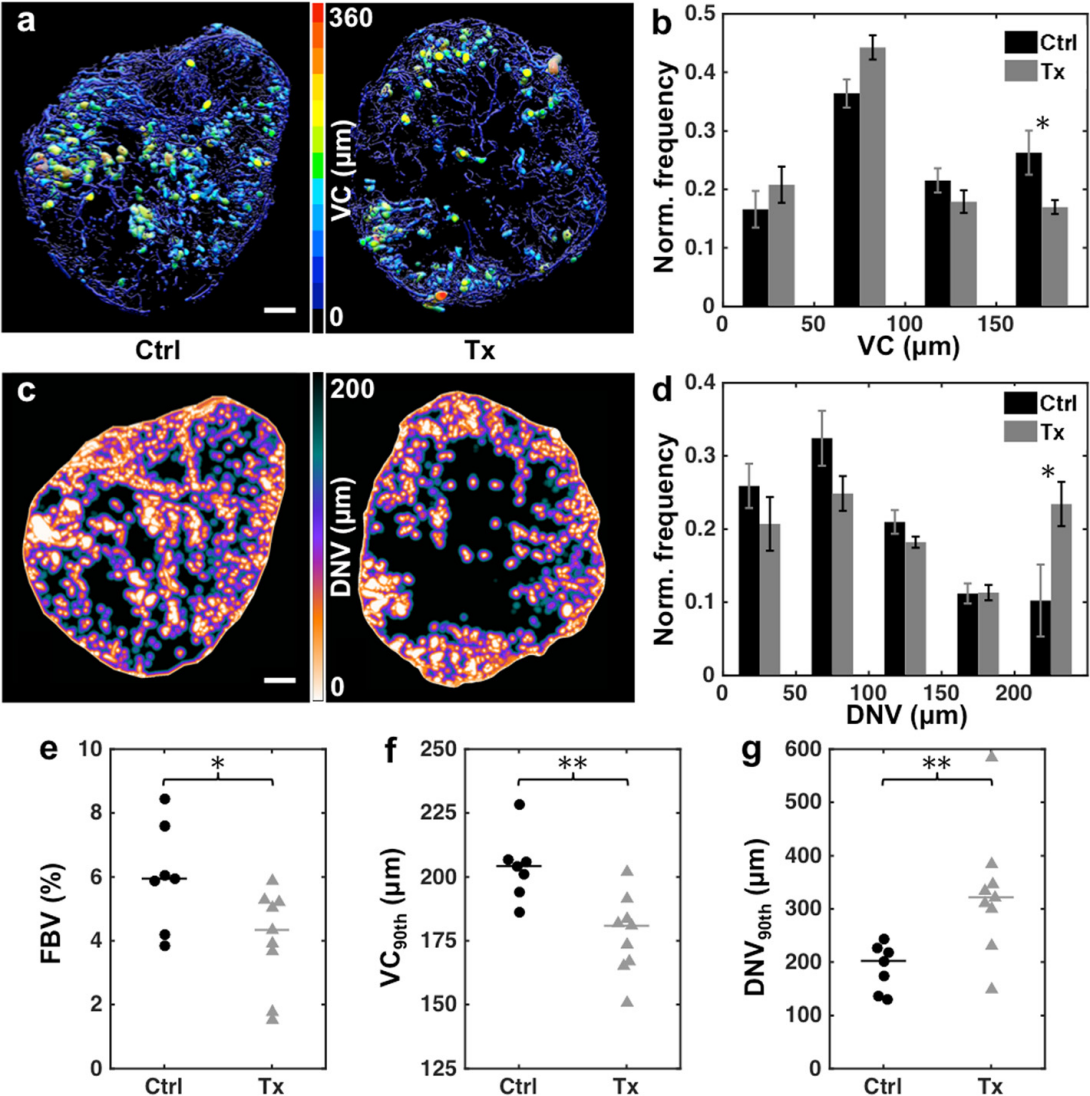
Ex vivo μCT reveals vascular response to cPLA2α inhibition. **a** Volume renderings of vasculature segmented from 150-slice sections of μCT images of representative control and treated tumors, color-coded by VC. Scale bar = 1 mm. **b** Normalized pooled histograms of VC in control and treated tumors. Bars indicate group medians, and error bars indicate median absolute deviations. **c** DNV maps corresponding to the central slices of the tumor sections depicted in **a**. Scale bar = 1 mm. **d** Normalized pooled histograms of DNV in control and treated tumors. Bars indicate group medians, and error bars indicate median absolute deviations. Dot plots of tumor-wise **e** FBV, **f** 90^th^ percentile VC, and **g** 90^th^ percentile DNV. Horizontal lines indicate group medians. * *p* < 0.05, ** *p* < 0.01, two-tailed Mann Whitney U test

To assess the effects of cPLA2α inhibition on tumor vascular morphology, ex vivo μCT was performed after in vivo MRI on day 7. Figure 4a shows volume renderings of the segmented μCT vasculature from 150-slice sections of representative control and treated tumors, with vessels color-coded by VC. As illustrated by the VC histograms (Fig. 4b), treated tumors had a smaller proportion of large vessels (VC > 150 μm) compared to control tumors: 0.170 ± 0.012 vs. 0.263 ± 0.038, *p* = 0.023. Concordantly, the 90^th^ percentile VC in treated tumors was significantly smaller than that in control tumors: 180.9 ± 10.4 μm vs. 204.3 ± 3.0 μm, *p* = 0.002 (Fig. 4f). The FBV was also significantly lower in treated vs. control tumors: 4.29 ± 0.86 % vs. 5.89 ± 1.63 %, *p* = 0.031 (Fig. 4e).

Figure 4c shows slices of the DNV maps from the corresponding tumors regions shown in Fig. 4a. It is readily apparent that the treated tumor has larger avascular regions than the control tumor. This is also the case at the group level, with a significantly larger fraction of tumor voxels in the treated group being more than 200 μm away from the nearest vessel (0.234 ± 0.030 vs. 0.102 ± 0.049, *p* = 0.016, Fig. 4d), which is the upper bound of the oxygen diffusion limit reported in the literature [23, 24]. The median and 90^th^ percentile DNV values were also greater in treated tumors compared to controls: 109.1 ± 11.8 μm vs. 92.2 ± 8.4 μm, *p* = 0.031; and 325.2 ± 49.0 μm vs. 221.4 ± 20.5 μm, *p* = 0.003, respectively (Fig. 4g).

### cPLA2α inhibition decreases endothelial cell proliferation

**Fig. 5:**
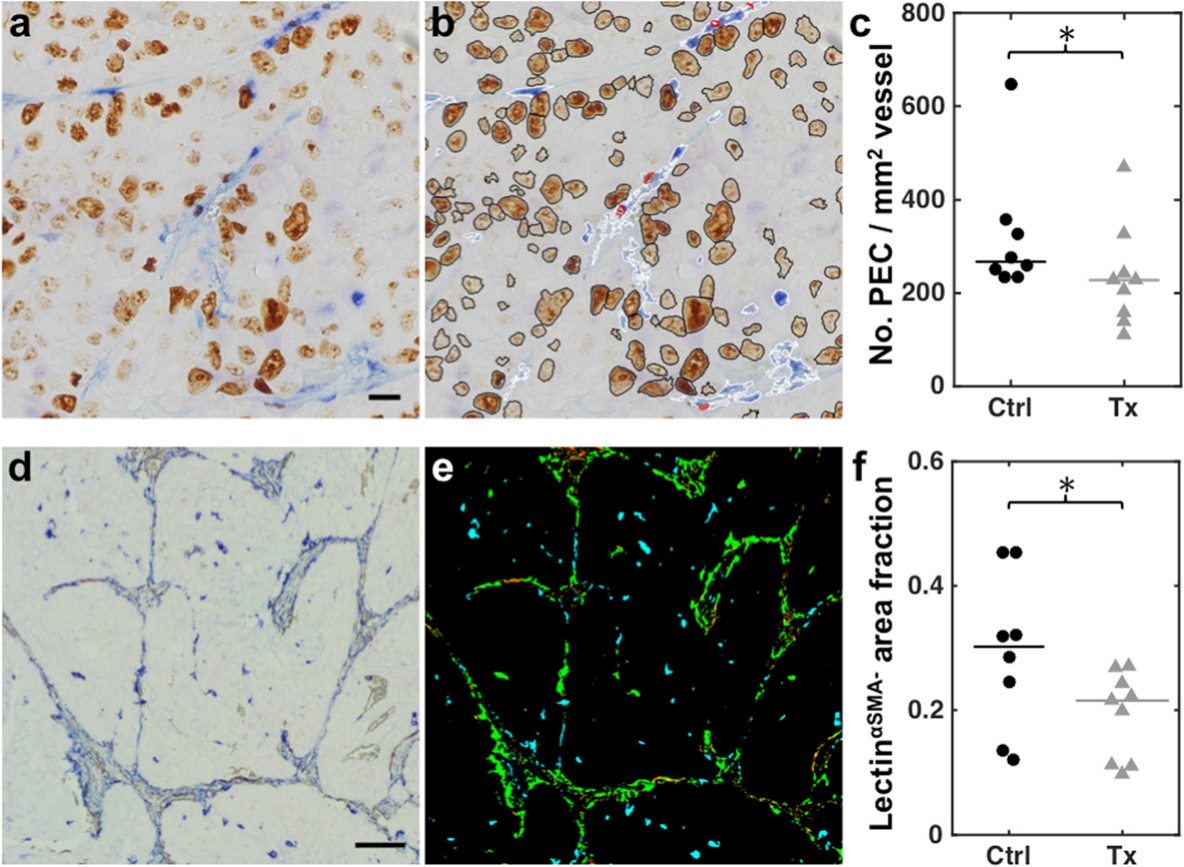
AVX235 reduces endothelial cell proliferation and targets immature vessels. **a** 40× image of a lectin- (*blue*) and Ki67-stained (*brown*) tumor section. Scale bar = 20 μm. **b** The same image showing the result of the automated segmentation. Lectin-stained blood vessels are outlined in *white*, Ki67-positive proliferating nuclei in *black*, and proliferating endothelial cells (PECs) in *red*. **c** Dot plot of the number of PECs per mm^2^ of blood vessel. Horizontal lines indicate group medians. **d** 10× image of a lectin- (*blue*) and α-SMA-stained (*brown*) tumor section. Scale bar = 100 μm. **e** Corresponding composite image of the automatically segmented α-SMA-positive (*red*, pericytes), lectin-positive (*green*, blood vessels), and lectin^α-SMA−^ (*blue*, immature vessels) regions. **f** Dot plot of the lectin^α-SMA−^ area fraction for each tumor. Horizontal lines indicate group medians. * *p* < 0.05, two-tailed Mann–Whitney U test

Lectin and Ki67 double staining was done to investigate the effect of AVX235 on cancer cell and endothelial cell proliferation. Figure 5a shows a representative 40× field of a lectin/Ki67 double-stained section, and Fig. 5b shows the result of the automatic segmentation, with lectin-stained vessels outlined in white, proliferating nuclei in black, and proliferating endothelial cell nuclei in red. There was no significant difference in the number of Ki67-positive proliferating cells between control and treated tumors at any time point (data not shown). After 7 days of treatment, a significantly lower number of proliferating endothelial cells normalized to vessel area was found in treated tumors compared to controls: 228 ± 68 per mm^2^ of vessel vs. 267 ± 32 per mm^2^ of vessel, *p* = 0.046 (Fig. 5c).

### AVX235-treated tumors contain fewer immature vessels

To estimate vessel maturity, we employed an anti-α-SMA antibody as a marker for perivascular mural cells. Figure 5d shows a representative 10× field of a lectin/α-SMA double-stained section. The corresponding segmented composite image (Fig. 5e) shows α-SMA-positive regions in the red channel, lectin-positive regions in the green channel, and lectin-positive regions not associated with α-SMA (lectin^α-SMA−^) in the blue channel. After 7 days of treatment, AVX235-treated tumors contained a lower fraction of lectin-stained endothelium that was not associated with α-SMA: 0.215 ± 0.053 vs. 0.302 ± 0.104, *p* = 0.036 (Fig. 5f). In other words, control tumors contained more vessels lacking pericyte coverage. There were no significant differences between control and treated tumors in the 2-day and 19-day groups.

## Discussion

A growing body of evidence implicates cPLA2α in the development of various cancers. Cytosolic PLA2 inhibition has previously been proven to suppress tumor growth and angiogenesis in preclinical cancer models [9, 11, 14]. BLBCs are known to be highly angiogenic and overexpress cPLA2α, making it a potential therapeutic target [4]. In this study, we characterized the anti-angiogenic effect of cPLA2α inhibition by AVX235 in patient-derived BLBC xenografts [4, 5]. To our knowledge, this study is the first to demonstrate therapeutic efficacy of cPLA2α inhibition in an in vivo breast cancer model.

PGE2 is produced from AA, which is released from membrane phospholipids by cPLA2α. The early treatment-induced reduction in tumor PGE2 levels suggests that AVX235 inhibited cPLA2α activity and could in part explain the effects of cPLA2α inhibition on tumor angiogenesis and growth.

PCho is a precursor of the cPLA2α substrate phosphatidylcholine (PtdCho); cPLA2α converts PtdCho to lysoPtdCho, which is further metabolized to GPC. The higher PCho and lower GPC levels in the treated samples are consistent with decreased cPLA2α activity. Choline-containing compounds are of special interest in cancer metabolism, and changes in their concentrations are associated with treatment response [29]. It was outside the scope of this study to determine whether these changes were directly connected to the anti-angiogenic response to AVX235, but it is possible that cPLA2α inhibition affects metabolic pathways that mediate signals to vascular cells.

Treatment with AVX235 led to significantly reduced tumor growth from day 8 onward. A previous study found a similar response to treatment with bevacizumab, a monoclonal antibody against VEGF-A, in the same model [30]. The bevacizumab-treated tumors displayed lower microvessel density and fewer proliferating endothelial cells; similarly, we found significantly decreased vascularization and endothelial cell proliferation in AVX235-treated tumors. As is commonly observed, significant anti-angiogenic effects preceded significant tumor growth inhibition. Since the proliferation of cancer cells was not affected by treatment, while proliferation of endothelial cells decreased, the growth inhibition likely resulted from anti-angiogenic, and not direct cytostatic, effects.

Tumor vasculature is characteristically abnormal, with irregular, disorganized, and leaky vessels. Anti-angiogenic therapies have been shown to induce a temporary normalization of tumor vasculature with an observable increase in perfusion, decrease in vessel permeability, and a shift towards more normal vessel morphology [31]. An early improvement in perfusion was seen in most AVX235-treated tumors, demonstrated by increases in the DCE-MRI parameters AUC_1min_ and FEV after 4 days of treatment. While not significantly different from what was observed in control tumors, this suggests that cPLA2α inhibition could have resulted in initial vascular normalization. An earlier study showed that bevacizumab treatment caused a similar initial increase in perfusion in the same tumor model [32].

Micro-CT showed that treated tumors contained fewer large, dilated vessels compared to controls. Reduction in tumor vessel caliber is a commonly reported response to anti-angiogenic therapies and also considered a sign of vascular normalization [33]. However, the decreases in AUC_1min_ and FEV between days 4 and 7 in treated tumors, consistent with the lower FBV and larger DNV (i.e., decreased vessel density) measured by μCT, indicate significant anti-vascular effects after 1 week of AVX235 therapy.

Improved pericyte coverage is another common normalizing effect of anti-angiogenic therapy [34]. Pericytes mechanically and functionally stabilize endothelial cells, and vessels that are not associated with pericytes are considered to be immature and less functional. There is no one universal molecular marker that identifies all pericytes (the definition of which is still debated), as the expression of the various markers may vary between pericytes [35]. A limitation of this study is the use of only one marker, thus some pericytes may not have been stained. But α-SMA is commonly used as a pericyte marker, and it is frequently upregulated in tumor pericytes [36]. In our model, we observed fewer immature vessels (i.e., vessels lacking α-SMA coverage) in AVX235 treated tumors. A previous study showed that cPLA2 may play an essential role in pericyte recruitment, demonstrated by the absence of α-SMA- and desmin-positive pericytes around tumor vasculature in cPLA2-deficient mice [14]. Therefore, it is unlikely that the reduced number of immature vessels following AVX235 administration was the result of increased pericyte coverage and vascular maturation, as has been reported in studies of anti-VEGF and other therapies [37–40]. Rather, our results imply that immature vessels lacking pericytes were pruned as a consequence of AVX235 treatment, or possibly that cPLA2α inhibition led to loss of pericytes with subsequent vessel regression.

The PGE2 levels, vessel density, vessel maturity, and number of proliferating cancer and endothelial cells all decreased at later time points independent of treatment (data not shown), indicating that the tumors changed phenotype with time, which has been demonstrated previously [23]. While the use of different cohorts and drug doses in the tumor growth study and imaging study complicates comparison between different time points, this phenotypic evolution may reflect a naturally developing insensitivity to anti-angiogenic therapy as the tumors become less angiogenic and more necrotic. The lack of significant metabolic and histological differences between control and treated tumors at day 19 could be due to the relatively small group sizes in this study or to sampling error associated with histological analysis. Alternatively, given that significant molecular differences were present at earlier time points, the absence of these same differences at day 19 may more likely be due to: 1) this particular model’s natural evolution toward a less angiogenic phenotype as the tumors grow larger [23], or 2) compensatory upregulation of alternative pathways in the tumors, which is a limitation to almost all targeted therapies [41]. Further investigation is necessary to test this hypothesis.

## Conclusions

Collectively, our data shows that AVX235 resulted in inhibition of cPLA2α activity, evidenced by decreases in the levels of key downstream metabolites, and in reduction in tumor vascularization and perfusion, which led to long-term tumor growth inhibition. As with other anti-angiogenic drugs, the therapeutic value of AVX235 in cancer would likely be maximized in a neoadjuvant setting, or in combination with conventional chemo- or radiotherapy. Ultimately, this study demonstrates that cPLA2 inhibitors could help address the need for better therapies for triple-negative basal-like breast cancer.

## Abbreviations

AA: arachidonic acid
AUC_1min_: initial area under the curve during first minute after contrast injection
BLBC: basal-like breast cancer
cPLA2α: group IVA cytosolic phospholipase A2
DCE-MRI: dynamic contrast enhanced magnetic resonance imaging
DNV: distance to nearest vessel
EIA: enzyme immunoassay
FBV: fractional blood volume
FEV: fraction of enhancing voxels
GPC: glycerophosphocholine
HR MAS MRS: high-resolution magic angle spinning magnetic resonance spectroscopy
NBF: neutral buffered formalin
PCho: phosphocholine
PEC: proliferating endothelial cell
PGE2: prostaglandin E2
PLS-DA: partial least squares discriminant analysis
PtdCho: phosphatidylcholine
RARE: rapid acquisition with relaxation enhancement
RSI_1min_: relative signal intensity 1 min after contrast injection
VC: vessel caliber
VIP: variable influence on projection
α-SMA: α-smooth muscle actin
μCT: micro-computed tomography

## References

[1] Nielsen TO, Hsu FD, Jensen K, Cheang M, Karaca G, Hu Z (2004). Immunohistochemical and clinical characterization of the basal-like subtype of invasive breast carcinoma immunohistochemical and clinical characterization of the basal- like subtype of invasive breast carcinoma. Clin Cancer Res.

[2] Sørlie T, Perou CM, Tibshirani R, Aas T, Geisler S, Johnsen H (2001). Gene expression patterns of breast carcinomas distinguish tumor subclasses with clinical implications. Proc Natl Acad Sci U S A.

[3] Bertucci F, Finetti P, Cervera N, Esterni B, Hermitte F, Viens P (2008). How basal are triple-negative breast cancers?. Int J Cancer.

[4] Grinde MT, Skrbo N, Moestue SA, Rødland EA, Borgan E, Kristian A, et al. Interplay of choline metabolites and genes in patient-derived breast cancer xenografts. Breast Cancer Res. 2014;16:R5. 10.1186/bcr3597 PMC3978476 24447408

[5] Moestue S a, Borgan E, Huuse EM, Lindholm EM, Sitter B, Børresen-Dale A-L, et al. Distinct choline metabolic profiles are associated with differences in gene expression for basal-like and luminal-like breast cancer xenograft models. BMC Cancer. 2010;10. 10.1186/1471-2407-10-433 PMC2931488 20716336

[6] Nakanishi M, Rosenberg DW (2006). Roles of cPLA2alpha and arachidonic acid in cancer. Biochim Biophys Acta.

[7] Wen Z-H, Su Y-C, Lai P-L, Zhang Y, Xu Y-F, Zhao A (2013). Critical role of arachidonic acid-activated mTOR signaling in breast carcinogenesis and angiogenesis. Oncogene.

[8] Patel MI, Singh J, Niknami M, Kurek C, Yao M, Lu S (2008). Cytosolic phospholipase A2-alpha: a potential therapeutic target for prostate cancer. Clin Cancer Res.

[9] Linkous A, Geng L, Lyshchik A, Hallahan DE, Yazlovitskaya EM (2009). Cytosolic phospholipase A2: targeting cancer through the tumor vasculature. Clin Cancer Res.

[10] Cai Q, Zhao Z, Antalis C, Yan L, Del Priore G, Hamed AH (2012). Elevated and secreted phospholipase A2 activities as new potential therapeutic targets in human epithelial ovarian cancer. FASEB J.

[11] Thotala D, Craft JM, Ferraro DJ, Kotipatruni RP, Bhave SR, Jaboin JJ (2013). Cytosolic phospholipaseA2 inhibition with PLA-695 radiosensitizes tumors in lung cancer animal models. PLoS One.

[12] Ghosh M, Tucker DE, Burchett SA, Leslie CC (2006). Properties of the group IV phospholipase A2 family. Prog Lipid Res.

[13] Yazlovitskaya EM, Linkous AG, Thotala DK, Cuneo KC, Hallahan DE (2008). Cytosolic phospholipase A2 regulates viability of irradiated vascular endothelium. Cell Death Differ.

[14] Linkous AG, Yazlovitskaya EM, Hallahan DE (2010). Cytosolic phospholipase A2 and lysophospholipids in tumor angiogenesis. J Natl Cancer Inst.

[15] Fujita Y, Yoshizumi M, Izawa Y, Ali N, Ohnishi H, Kanematsu Y, et al. Transactivation of fetal liver kinase-1/kinase-insert domain-containing receptor by lysophosphatidylcholine induces vascular endothelial cell proliferation. Endocrinology. 2006;147:1377–85. 10.1210/en.2005-0644 16322069

[16] Wang D, Dubois RN (2010). Eicosanoids and cancer. Nat Rev Cancer.

[17] Greenhough A, Smartt HJM, Moore AE, Roberts HR, Williams AC, Paraskeva C (2009). The COX-2/PGE2 pathway: Key roles in the hallmarks of cancer and adaptation to the tumour microenvironment. Carcinogenesis.

[18] Chen EP, Smyth EM (2011). COX-2 and PGE2-dependent immunomodulation in breast cancer. Prostaglandins Other Lipid Mediat.

[19] Timoshenko AV, Xu G, Chakrabarti S, Lala PK, Chakraborty C (2003). Role of prostaglandin E2 receptors in migration of murine and human breast cancer cells. Exp Cell Res.

[20] Chang S-H, Liu CH, Conway R, Han DK, Nithipatikom K, Trifan OC (2004). Role of prostaglandin E2-dependent angiogenic switch in cyclooxygenase 2-induced breast cancer progression. Proc Natl Acad Sci U S A.

[21] Bergamaschi A, Hjortland GO, Triulzi T, Sørlie T, Johnsen H, Ree AH (2009). Molecular profiling and characterization of luminal-like and basal-like in vivo breast cancer xenograft models. Mol Oncol.

[22] Kokotos G, Feuerherm AJ, Barbayianni E, Shah I, Sæther M, Magrioti V (2014). Inhibition of group IVA cytosolic phospholipase A2 by thiazolyl ketones in vitro, ex vivo, and in vivo. J Med Chem.

[23] Huuse EM, Moestue S a, Lindholm EM, Bathen TF, Nalwoga H, Krüger K (2012). In vivo MRI and histopathological assessment of tumor microenvironment in luminal-like and basal-like breast cancer xenografts. J Magn Reson Imaging.

[24] Wold S, Sjöström M, Eriksson L (2001). PLS-regression: A basic tool of chemometrics. Chemometrics and Intelligent Laboratory Systems.

[25] Chong IG, Jun CH (2005). Performance of some variable selection methods when multicollinearity is present. Chemom Intell Lab Syst.

[26] Schindelin J, Arganda-Carreras I, Frise E, Kaynig V, Longair M, Pietzsch T, et al. Fiji: an open source platform for biological image analysis. Nat Methods. 2012;9:676–82. 10.1038/nmeth.2019 PMC3855844 22743772

[27] Kim E, Cebulla J, Ward BD, Rhie K, Zhang J, Pathak AP (2013). Assessing breast cancer angiogenesis in vivo: Which susceptibility contrast MRI biomarkers are relevant?. Magn Reson Med.

[28] Westerhuis J a, Hoefsloot HCJ, Smit S, Vis DJ, Smilde AK, van Velzen EJJ, et al. Assessment of PLSDA cross validation. Metabolomics. 2008;4:81–9.

[29] Jagannathan NR, Kumar M, Seenu V, Coshic O, Dwivedi SN, Julka PK (2001). Evaluation of total choline from in-vivo volume localized proton MR spectroscopy and its response to neoadjuvant chemotherapy in locally advanced breast cancer. Br J Cancer.

[30] Lindholm EM, Kristian A, Nalwoga H, Krüger K, Nygård S, Akslen L a (2012). Effect of antiangiogenic therapy on tumor growth, vasculature and kinase activity in basal- and luminal-like breast cancer xenografts. Mol Oncol.

[31] Carmeliet P, Jain RK (2011). Principles and mechanisms of vessel normalization for cancer and other angiogenic diseases. Nat Rev Drug Discov.

[32] Moestue S a, Huuse EM, Lindholm EM, Bofin A, Engebraaten O, Mælandsmo GM (2013). Low-molecular contrast agent dynamic contrast-enhanced (DCE)-MRI and diffusion-weighted (DW)-MRI in early assessment of bevacizumab treatment in breast cancer xenografts. J Magn Reson Imaging.

[33] Emblem KE, Farrar CT, Gerstner ER, Batchelor TT, Borra RJH, Rosen BR (2014). Vessel calibre-a potential MRI biomarker of tumour response in clinical trials. Nat Rev Clin Oncol.

[34] Goel S, Duda DG, Xu L, Munn LL, Boucher Y, Fukumura D (2011). Normalization of the vasculature for treatment of cancer and other diseases. Physiol Rev.

[35] Armulik A, Genové G, Betsholtz C (2011). Pericytes: Developmental, physiological, and pathological perspectives, problems, and promises. Dev Cell.

[36] Morikawa S, Baluk P, Kaidoh T, Haskell A, Jain RK, McDonald DM (2002). Abnormalities in pericytes on blood vessels and endothelial sprouts in tumors. Am J Pathol.

[37] Dickson PV, Hamner JB, Sims TL, Fraga CH, Ng CYC, Rajasekeran S (2007). Bevacizumab-induced transient remodeling of the vasculature in neuroblastoma xenografts results in improved delivery and efficacy of systemically administered chemotherapy. Clin Cancer Res.

[38] Tong RT, Boucher Y, Kozin SV, Winkler F, Hicklin DJ, Jain RK (2004). Vascular normalization by vascular endothelial growth factor receptor 2 blockade induces a pressure gradient across the vasculature and improves drug penetration in tumors. Cancer Res.

[39] Bhattacharya A, Seshadri M, Oven SD, Tóth K, Vaughan MM, Rustum YM (2008). Tumor vascular maturation and improved drug delivery induced by methylselenocysteine leads to therapeutic synergy with anticancer drugs. Clin Cancer Res.

[40] Tian S, Hayes a J, Metheny-Barlow LJ, Li L-Y (2002). Stabilization of breast cancer xenograft tumour neovasculature by angiopoietin-1. Br J Cancer.

[41] Ellis LM, Hicklin DJ (2009). Resistance to targeted therapies: Refining anticancer therapy in the era of molecular oncology. Clin Cancer Res.

